# Review of Evidence Suggesting That the Fascia Network Could Be the Anatomical Basis for Acupoints and Meridians in the Human Body

**DOI:** 10.1155/2011/260510

**Published:** 2011-04-26

**Authors:** Yu Bai, Jun Wang, Jin-peng Wu, Jing-xing Dai, Ou Sha, David Tai Wai Yew, Lin Yuan, Qiu-ni Liang

**Affiliations:** ^1^Department of Anatomy, Southern Medical University, Guangzhou 510515, China; ^2^Department of Anatomy, School of Medicine of Shenzhen University, Shenzhen 518060, China; ^3^Department of Anatomy, The Chinese University of Hong Kong, Shatin, Hong Kong

## Abstract

The anatomical basis for the concept of meridians in traditional Chinese medicine (TCM) has not been resolved. This paper reviews the evidence supporting a relationship between acupuncture points/meridians and fascia. The reviewed evidence supports the view that the human body's fascia network may be the physical substrate represented by the meridians of TCM. Specifically, this hypothesis is supported by anatomical observations of body scan data demonstrating that the fascia network resembles the theoretical meridian system in salient ways, as well as physiological, histological, and clinical observations. This view represents a theoretical basis and means for applying modern biomedical research to examining TCM principles and therapies, and it favors a holistic approach to diagnosis and treatment.

## 1. Introduction

**Figure 1:**
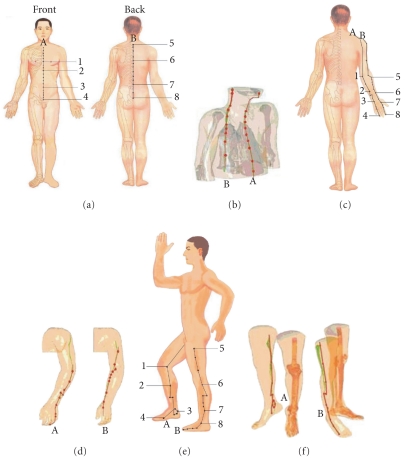
Comparison between acupuncture points (from the anatomical guidelines provided in [19]) and fascia imaging data. The fascia connective tissue gathering areas were constructed using MIMICS11.02 software based on the digital datasets of VCH bodies. (a) Location of the Ren Channel (A) and Du Channel (B): (1) Tanzhong. (2) Juque. (3) Shenque. (4) Qugu. (5) Dazhui. (6) Shendao. (7) Yaoyangguan. (8) Yaoshu. (b) Reconstruction of fascia pathways approximating the Ren Channel (A) and Du Channel (B). (c) Locations of the Triple energizer meridian of hand-shaoyang (a) and the Large intestine meridian of hand-yangming (b): (1) Tianjing. (2) Sanyangluo. (3) Yangchi. (4) Guanchong. (5) Quchi. (6) Pianli. (7) Hegu. (8) Shangyang. (d) Reconstruction of fascia pathways in the arm approximating the Triple energizer meridian of hand-shaoyang (A) and the Large intestine meridian of hand-yangming (B). (e) Locations of the Kidney meridian of foot-shaoyin (A) and the Gallbladder meridian of foot-shaoyang (B): (1) Yingu. (2) Zhubin. (3) Dazhong. (4) Rangu. (5) Fengshi. (6) Yanglingquan. (7) Xuanzhong. (8) Qiuxu. (f) Reconstruction of fascia pathways in the leg approximating the Kidney meridian of foot-shaoyin (A) and the Gallbladder meridian of foot-shaoyang (B).

The theory of meridians and collaterals, also popularly known as channel theory, is a fundamental pillar of traditional Chinese medicine (TCM), particularly in the areas of acupuncture, moxibustion, and massage, as well as of traditional martial arts such as Tai Chi Chuan [1, 2]. In principle, meridians (jīngluò in Chinese) are essentially strings of acupoints, which may be visualized as passageways through which energy flows throughout the body [2, 3]. The meridian system is thought to be composed of 12 principal meridians, each of which connects to an organ system and extends to an extremity, and eight collaterals (Figures 1(a), 1(c), and 1(e)). Practitioners of TCM intend that their treatments should improve the flow of energy through the meridian network.

Given the long history of channel theory, which predates modern scientific development, and the theory's intermingling with philosophy and ancient metaphysical ideas, rigorous scientific and clinical studies are needed to tease out their true, physical nature [2]. Acupuncture is an ancient aspect of TCM with demonstrated therapeutic effects [2]. Although scientific interest in the validity of meridians and acupoints has been growing in the last decade, the basis of the nature and material of acupuncture points and meridians has not been resolved. Lo proposed that acupuncture meridians are made up of polarized molecules [4]. Based on a review of the literature, Ahn et al. concluded that the available evidence did not conclusively support the claim that acupuncture points had distinct electrical properties [5]. Ma and colleagues have speculated that some aspect of the perivascular space might be the anatomical substrate of meridians [6]. Li et al. reported evidence suggesting that visualized regional hypodermic migration channels of interstitial fluid constituted the meridians [7]. Other research groups have since provided complementary supportive evidence with various approaches for this acupoint simulation-brain activation phenomenon [8–11].

While evidence consistent with the existence of entities termed meridians is growing, much work is still needed to delineate their anatomical basis [12]. It has been posited that the physical substrate of meridians may include neurovascular bundles, neuromuscular attachments, sensory nerve endings, perivascular space and perineurial vessels [6, 13–17]. In particular, Langevin and Yandow proposed that the anatomical relationship of acupuncture points and meridians to connective tissue planes [18].

Based on supportive evidence in the literature, the present paper provides support for a fascia network hypothesis of meridians—that is, the view that the fascia network may be the anatomical basis for acupoints and meridians in the human body. Specifically, we examine whether the evidence supports the ideas that (i) the anatomical basis of meridians is the fascia network that is distributed throughout the body and (ii) the histological composition of meridians is nonspecific connective tissues, including loose connective tissue and fat tissue. The histological structures where an acupuncture needle acts are fascia connective tissue containing nerve endings, capillary vessels, fibroblasts, undifferentiated mesenchymal cells, lymphocytes, and so forth. Acupuncture points are traditionally believed to be sites that produce strong reactions when stimulated. The distribution of fascia connective tissue throughout the body enables acupoints to exist in every part of the body. In our view, the difference between clinically recognized acupoints and nonacupoints, as well as between main acupoints and supplementary acupoints, lies principally in the intensity of biological reactions rather than in the gross structural components *per se*. Below, our view that the fascia may be the physical substrate of the meridian system is evaluated with respect to prior anatomical observations of body scan data demonstrating that three-dimensional (3D) reconstructions of fascia resemble the theoretical meridian system in salient ways and relevant physiological observations. 

## 2. Anatomical and Physiological Observations

### 2.1. Human Body Imaging

**Figure 2:**
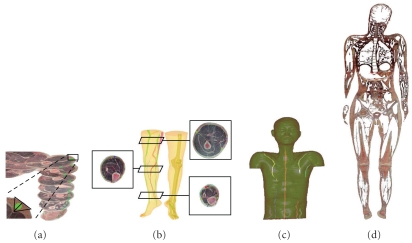
Thick fascia connective tissues in VCH images were marked (green) (a) and their 3 D structures were rendered (b, c). When fascia connective tissues of the whole body, including thick and thin fascia tissues, were marked and their 3 D structures were reconstructed, a complete fascia network was observed; (d see reference no. [19]). All of the human organs and tissues were observed to be coated with connective tissues, and the connective tissues extended into the organs to form septa within the organs.

The 3D constructions of the human body produced by the visible Chinese human (VCH) project, a National Basic Research Program of China, provide an intriguing window into human anatomy [19, 20]. Indeed, as illustrated in Figure 1, scan data show that the fascial connective tissues of the human body approximate conspicuously the TCM meridian network [19, 20]. Firstly, it is noteworthy that reconstructions of the fascial connective tissues in the body trunk and limbs show line-like structures which are similar to those of acupoints and meridians/collaterals [19]. Secondly, these fascial strings form a network of lines that are close to the virtual meridians in anatomical location [19]. Furthermore, as shown in Figure 2, subsequent 3D fascial reconstructive studies involving computed tomography and magnetic resonance imaging (MRI) of living human bodies revealed a pattern of line-like structures that appear similar in form and distribution to the traditional Chinese meridians [19, 20]. The VCH and living body imaging studies together indicate that the anatomy of the fascial network in the human body is consistent with the traditional view of the meridian network pattern. 

### 2.2. Acupuncture

While Cho et al.'s imaging data suggesting a possible connection between acupuncture and brain activation discussed above provide evidence that meridians should have a physical anatomical substrate, they did not provide precise information regarding what that substrate may be [21, 22]. Studies of the local acupuncture process itself may shed light on the anatomical substrate. Efficacious acupuncture is associated with a temporary local sensation of soreness and/or numbness (termed deqi) at the acupoint site [23]. The needle grasp phenomenon has been shown to occur when a needle physically impacts the connective tissue in the fascia [18, 24]. This observation indicates that the efficacy of acupuncture relies on interaction with the fascia. 

### 2.3. Physiological Observations

Fascia is the soft tissue component of the connective tissue system that permeates the human body. It forms a whole-body continuous 3D matrix of structural support [25]. It penetrates and surrounds all of the body's vital organs, muscles, bones, and nerve fibers, creating a unique physiological environment [25]. This network of fascial connective tissue is situated to provide ongoing physiological support for the body's metabolically active systems composed of specialized cells and tissues [25]. In the view of TCM, optimal health requires unencumbered flow of energy through the meridians. Of course, TCM does not specify the physical nature of such “energy.” If the meridians are fascia, as we posit, then that energy may be nerve signals, flow of paracrine signaling molecules, electrical signaling through gap junctions among perineurial cells, distribution of mechanical forces, or some combination of these processes. 

### 2.4. Fascia Provides Dynamic Connections between and among Muscles and Bones

Van der Wal used 3D reconstruction studies to reveal a continuous connective tissue structure that runs throughout the body allowing for dynamic connections between the fascia and musculature [26]. Van der Wal's work was revolutionary in that it has provided a view of the fascia as an integrated structure, rather than distinct piecemeal structures associated with particular muscles and/or bones [26]. The integrated nature of the fascia is consistent with its presently hypothesized role as the meridian network of the body. 

In a study examining the flexor carpi ulnaris in human patients undergoing tendon transfer surgery, Smeulders and Kreulen found that the intermuscular connections, rather than individual tendon-muscle connections, were responsible for most (90%) of the transference of forces between the neighboring muscles [27]. These researchers further found that muscle excursion was more limited by intermuscular connective tissues than by tendon-muscle connections [27]. Indeed, Huijing and Baan showed that as much as half of the force generated by a muscle is transmitted to surrounding connective tissues [28]. Thus, it appears that the fasciae mediate an active mechanical transference role. 

### 2.5. Responsivity of Cells to Tensional Forces

The utility of mechanical force activation of neuronal receptors has long been appreciated for touch and pressure sensation as well as for protection signaling of potential injury due to hyperextension, hypercompression, or hyperrotation. Indeed integrin receptors expressed in the extracellular matrix are mechanically coupled to intra-cellular actin filaments, the contortion of which can initiate biochemical signaling resulting in adaptive changes in cell morphology [29]. Hence, mechano-sensation in connective tissues does not merely passively relay information to the central nervous system, but also directly impacts the properties of the fascia itself including its fibroblasts and collagen fibers [30–32]. Furthermore, Grinnell demonstrated that cells do not adhere indiscriminately to matrix proteins, but rather adhere to specific matrix fibrils, supporting the notion of an organized function fascial network [33]. Fascia inflammation will be addressed in the following section, but it is worth mentioning here that repetitive mechanical strain was experimentally shown to affect secretion of proinflammatory interleukins and cell proliferation of human fibroblasts *in vitro *[34, 35]. 

### 2.6. Perineurium and Epineurium Implicated in Pain and Inflammation

The fascia tissue surrounding nerve fascicles (the perineurium) and that surrounding the whole nerve and associated vasculature bundles (the epineurium) play important roles in pain regulation [36]. Bove and Light conducted immunohistochemistry studies revealing peptidergic fine-caliber axons in the epineurium and perineurium that are consistent with nociceptive function [37]. Subsequent *in vitro* electrical and chemical nerve stimulation studies demonstrated that stimulation of local nociceptive receptors of the perineurium and/or epineurium can evoke neurogenic inflammation [38]. Such evidence indicates that disruption of perineural fascial tissues that stimulates perineural nociceptors can trigger local (neurogenic) inflammation, presumably as a defensive mechanism that functions to help maintain the local environment of the nerves [39].

Indeed a convergence of evidence indicates that chronic low back pain may be emanating from connective tissues, rather than bone, cartilage, or musculature. The magnitude of low back pain was found not to correlate with magnitude of disc displacement [40]. Subsequent work has implicated perispinal ligamentous tissues and lumbar fascia as common culprits of low back pain [41, 42]. Moreover, Thomas and Robet found corroborating histological evidence indicating that low back pain may be due to inflammation in the lumbar fasciae [43]. Based on a convergence of evidence, such as the above studies among others, Langevin and Sherman have developed a model of chronic low back pain in which the pain is the result of a cycle of protective reduced immobility leading to fascial remodeling, resulting in inflammation and neural sensitization, which then further restrain mobility and perpetuate the cycle [44]. The phenomenon of neurogenic inflammation triggered by stimulation of nociceptive receptors in fascial tissues is consistent with the notion that disruption of fascial physiology can have notable consequences on human health. 

## 3. Discussion

In this paper, a convergence of evidence from various fields related to fascial anatomy and physiology were reviewed and considered with respect to the possibility that the fascia might be the physical substrate referred to as the meridian system in TCM. The anatomy of the fascial network in the human body, as demonstrated through VCH and living body imaging studies, is consistent with the traditional view of the meridian network pattern, and the efficacy of acupuncture has been shown to rely on interactions with the fascia. Additionally, it appears that the fasciae mediate an active mechanical transference role as they provide dynamic connections between and among the muscles and bones. Moreover, the phenomenon of neurogenic inflammation triggered by stimulation of nociceptive receptors in fascial tissues is consistent with the notion that disruption of fascial physiology can have notable consequences on human health. Indeed, it is our view that neurogenic inflammation in fasciae may constitute a form of disruption of meridian energy flow in TCM. 

If the fascia network of the body is indeed the physical substrate of the meridians of TCM, there are important clinical and research implications. Specifically, if evidence continues to mount in support of this view, then the fasciae should receive greater attention in both diagnostics and treatment [45]. An important ramification of fascial meridians is that this view favors a more holistic approach to medicine, in which the body's interconnections and interactions are considered [45]. Further research resolving the neurophysiology of perineural receptors and facial architecture should help inform therapies for chronic pain, spasticity, and perhaps other thus far poorly understood idiopathic conditions.

Considering fascia as the physical substrate of the meridians of TCM has fundamental ramifications for biomedical research as well. The meridian view of fascia can provide a theoretical basis and means for applying modern biomedical research to examining TCM principles and therapies. Specifically, if true, then contributions of the fascia to ongoing metabolism support and ultimately long-term health and longevity should be observable. Also, if true, then the fascia should provide a regenerative resource for the body, perhaps as a source of stem cells and progenitor cells [46]. The physiological support and progenerative role of the fascia may emerge early in development; if so, its contributions during embryonic tissue differentiation should receive greater attention. Perhaps, in the future, more in-depth study of the fascial network could form a new discipline, namely, fasciaology. 

## References

[1] Birch SJ, Felt RL (1999). The theoretical basis of acupuncture: fundamental concepts and explanatory models. *Understanding Acupuncture*.

[2] Xutian S, Zhang J, Louise W (2009). New exploration and understanding of traditional Chinese medicine. *American Journal of Chinese Medicine*.

[3] Sutherland JA (2000). Meridian therapy: current research and implications for critical care. *AACN Clinical Issues*.

[4] Lo SY (2002). Meridians in acupuncture and infrared imaging. *Medical Hypotheses*.

[5] Ahn AC, Colbert AP, Anderson BJ (2008). Electrical properties of acupuncture points and meridians: a systematic review. *Bioelectromagnetics*.

[6] Ma W, Tong H, Xu W (2003). Perivascular space: possible anatomical substrate for the meridian. *Journal of Alternative and Complementary Medicine*.

[7] Li HY, Yang JF, Chen M (2008). Visualized regional hypodermic migration channels of interstitial fluid in human beings: are these ancient meridians?. *Journal of Alternative and Complementary Medicine*.

[8] Lee H, Park HJ, Kim SA (2002). Acupuncture stimulation of the vision-related acupoint (Bl-67) increases c-Fos expression in the visual cortex of binocularly deprived rat pups. *American Journal of Chinese Medicine*.

[9] Siedentopf CM, Golaszewski SM, Mottaghy FM, Ruff CC, Felber S, Schlager A (2002). Functional magnetic resonance imaging detects activation of the visual association cortex during laser acupuncture of the foot in humans. *Neuroscience Letters*.

[10] Li G, Cheung RTF, Ma QY, Yang ES (2003). Visual cortical activations on fMRI upon stimulation of the vision-implicated acupoints. *NeuroReport*.

[11] Litscher G, Rachbauer D, Ropele S (2004). Acupuncture using laser needles modulates brain function: first evidence from functional transcranial Doppler sonography and functional magnetic resonance imaging. *Lasers in Medical Science*.

[12] Longhurst JC (2010). Defining meridians: a modern basis of understanding. *JAMS Journal of Acupuncture and Meridian Studies*.

[13] Bossy J (1984). Morphological data concerning the acupuncture points and channel network. *Acupuncture and Electro-Therapeutics Research*.

[14] Dung HC (1984). Anatomical features contributing to the formation of acupuncture points. *American Journal of Acupuncture*.

[15] Ciszek M, Szopinski J, Skrzypulec V (1985). Investigations of morphological structure of acupuncture points and meridians. *Journal of Traditional Chinese Medicine*.

[16] Hashimoto PH (2005). The perineurial vessel: a possible candidate for the structural basis of the meridian (Jing-Lyo) in Chinese medicine. *Anatomical Science International*.

[17] Yung KT (2005). A birdcage model for the Chinese meridian system: part VI. Meridians as the primary regulatory system. *American Journal of Chinese Medicine*.

[18] Langevin HM, Yandow JA (2002). Relationship of acupuncture points and meridians to connective tissue planes. *Anatomical Record*.

[19] Wang J, Dong WR, Wang CL (2007). From meridians and acupoints to self-supervision and control system: a hypothesis of the 10th functional system based on anatomical studies of digitized virtual human. *Journal of Southern Medical University*.

[20] Wang CL, Yuan L, Wang J, Jiao PF Contrast study on the line course of fascia meridians made by three dimensional reconstruction and classical meridians in human body. *Chinese Journal of Anatomy*.

[21] Cho ZH, Chung SC, Jones JP (1998). New findings of the correlation between acupoints and corresponding brain cortices using functional MRI. *Proceedings of the National Academy of Sciences of the United States of America*.

[22] Cho ZH, Chung SC, Lee HJ, Wong EK, Min BI (2006). Retraction. New findings of the correlation between acupoints and corresponding brain cortices using functional MRI. *Proceedings of the National Academy of Sciences of the United States of America*.

[23] Hui KKS, Nixon EE, Vangel MG (2007). Characterization of the “deqi” response in acupuncture. *BMC Complementary and Alternative Medicine*.

[24] Konofagou EE, Langevin HM (2005). Using ultrasound to understand acupuncture. Acupuncture needle manipulation and its effect on connective tissue. *IEEE Engineering in Medicine and Biology Magazine*.

[25] Thomas F, Robet S (2009). Introduction. *Fascia Research II, Amsterdam Basic Science and Implications for Conventional and Complementary Health Care*.

[26] van der Wal J (2009). The Architecture of the connective tissue in the musculoskeletal system—an often overlooked functional parameter as to proprioception in the locomotor apparatus. *Fascia Research II, Amsterdam Basic Science and Implications for Conventional and Complementary Health Care*.

[27] Smeulders MJC, Kreulen M (2007). Myofascial force transmission and tendon transfer for patients suffering from spastic paresis: a review and some new observations. *Journal of Electromyography and Kinesiology*.

[28] Huijing PA, Baan GC (2003). Myofascial force transmission: muscle relative position and length determine agonist and synergist muscle force. *Journal of Applied Physiology*.

[29] Thomas F, Robet S (2009). Introduction. *Fascia Research II, Amsterdam Basic Science and Implications for Conventional and Complementary Health Care*.

[30] Langevin HM, Bouffard NA, Badger GJ, Iatridis JC, Howe AK (2005). Dynamic fibroblast cytoskeletal response to subcutaneous tissue stretch ex vivo and in vivo. *American Journal of Physiology*.

[31] Langevin HM, Cornbrooks CJ, Taatjes DJ (2004). Fibroblasts form a body-wide cellular network. *Histochemistry and Cell Biology*.

[32] Yu X, Ding G, Huang H, Lin J, Yao W, Zhan R (2009). Role of collagen fibers in acupuncture analgesia therapy on rats. *Connective Tissue Research*.

[33] Grinnell F (2008). Fibroblast mechanics in three-dimensional collagen matrices. *Journal of Bodywork and Movement Therapies*.

[34] Meltzer KR, Standley PR (2007). Modeled repetitive motion strain and indirect osteopathic manipulative techniques in regulation of human fibroblast proliferation and interleukin secretion. *Journal of the American Osteopathic Association*.

[35] Eagan TS, Meltzer KR, Standley PR (2007). Importance of strain direction in regulating human fibroblast proliferation and cytokine secretion: a useful in vitro model for soft tissue injury and manual medicine treatments. *Journal of Manipulative and Physiological Therapeutics*.

[36] Bove GM (2008). Epi-perineurial anatomy, innervation, and axonal nociceptive mechanisms. *Journal of Bodywork and Movement Therapies*.

[37] Bove GM, Light AR (1995). Calcitonin gene-related peptide and peripherin immunoreaetivity in nerve sheaths. *Somatosensory and Motor Research*.

[38] Sauer SK, Bove GM, Averbeck B, Reeh PW (1999). Rat peripheral nerve components release calcitonin gene-related peptide and prostaglandin E in response to noxious stimuli: evidence that nervi nervorum are nociceptors. *Neuroscience*.

[39] Light AR (2004). “Nocifensor” system re-revisited. Focus on “two types of C nociceptor in human skin and their behavior in areas of capaicin-induced secondary hyperalgesia”. *Journal of Neurophysiology*.

[40] Jensen MC, Brant-Zawadzki MN, Obuchowski N, Modic MT, Malkasian D, Ross JS (1994). Magnetic resonance imaging of the lumbar spine in people without back pain. *The New England Journal of Medicine*.

[41] Panjabi MM (2006). A hypothesis of chronic back pain: ligament subfailure injuries lead to muscle control dysfunction. *European Spine Journal*.

[42] Schleip R, Vleeming A, Lehmann-Horn F, Klingler W (2007). Letter to the Editor concerning “A hypothesis of chronic back pain: ligament subfailure injuries lead to muscle control dysfunction” (M. Panjabi). *European Spine Journal*.

[43] Thomas F, Robet S (2009). Introduction. *Fascia Research II, Amsterdam Basic Science and Implications for Conventional and Complementary Health Care*.

[44] Langevin HM, Sherman KJ (2006). Pathophysiological model for chronic low back pain integrating connective tissue and nervous system mechanisms. *Medical Hypotheses*.

[45] Langevin HM (2006). Connective tissue: a body-wide signaling network?. *Medical Hypotheses*.

[46] Tapp H, Hanley EN, Patt JC, Gruber HE (2009). Adipose-derived stem cells: characterization and current application in orthopaedic tissue repair. *Experimental Biology and Medicine*.

